# Impact of Processed Food (Canteen and Oil Wastes) on the Development of Black Soldier Fly (*Hermetia illucens*) Larvae and Their Gut Microbiome Functions

**DOI:** 10.3389/fmicb.2021.619112

**Published:** 2021-01-21

**Authors:** Thomas Klammsteiner, Andreas Walter, Tajda Bogataj, Carina D. Heussler, Blaž Stres, Florian M. Steiner, Birgit C. Schlick-Steiner, Heribert Insam

**Affiliations:** ^1^Department of Microbiology, University of Innsbruck, Innsbruck, Austria; ^2^Department of Biotechnology and Food Engineering, MCI – The Entrepreneurial School, Innsbruck, Austria; ^3^Department of Ecology, University of Innsbruck, Innsbruck, Austria; ^4^Department of Animal Science, University of Ljubljana, Ljubljana, Slovenia; ^5^Institute of Sanitary Engineering, University of Ljubljana, Ljubljana, Slovenia; ^6^Faculty of Medicine, University of Ljubljana, Ljubljana, Slovenia

**Keywords:** animal feedstuff, waste valorization, circular economy, metabolism, microbial communities, 16S amplicon sequencing, oil waste, growth parameters

## Abstract

Canteens represent an essential food supply hub for educational institutions, companies, and business parks. Many people in these locations rely on a guaranteed service with consistent quality. It is an ongoing challenge to satisfy the demand for sufficient serving numbers, portion sizes, and menu variations to cover food intolerances and different palates of customers. However, overestimating this demand or fluctuating quality of dishes leads to an inevitable loss of unconsumed food due to leftovers. In this study, the food waste fraction of canteen leftovers was identified as an optimal diet for black soldier fly (*Hermetia illucens*) larvae based on 50% higher consumption and 15% higher waste reduction indices compared with control chicken feed diet. Although the digestibility of food waste was nearly twice as high, the conversion efficiency of ingested and digested chicken feed remains unparalleled (17.9 ± 0.6 and 37.5 ± 0.9 in CFD and 7.9 ± 0.9 and 9.6 ± 1.0 in FWD, respectively). The oil separator waste fraction, however, inhibited biomass gain by at least 85% and ultimately led to a larval mortality of up to 96%. In addition to monitoring larval development, we characterized physicochemical properties of pre- and post-process food waste substrates. High-throughput amplicon sequencing identified Firmicutes, Proteobacteria, and Bacteroidota as the most abundant phyla, and *Morganella*, *Acinetobacter*, and certain Lactobacillales species were identified as indicator species. By using metagenome imputation, we additionally gained insights into the functional spectrum of gut microbial communities. We anticipate that the results will contribute to the development of decentralized waste-management sites that make use of larvae to process food waste as it has become common practice for biogas plants.

## Introduction

With a more fast-moving society in industrialized countries, amounts of food waste in these countries as large as the total net food production of sub-Saharan Africa come along (FAO, 2011). In addition, a recent study by Verma et al. (2020) indicates that the worldwide food waste might be even twice as high than previous statistics published by the Food and Agriculture Organization of the United Nations FAO (2011). In contrast to food loss that occurs early in the food supply chain due to a decrease in quality and improper handling, the extent of food waste originates in the consumers’ values, behavior, and attitude (Principato, 2018). This mindset is also reflected by food waste accumulating in food service outlets such as canteens, cafeterias, and buffets. Canteens represent an important source of food waste in hubs of human productivity and many parameters influencing the extent of leftovers have been identified in the past: a lack of flexibility in adapting to consumer preferences, excessive portion sizes, displeasing consumption settings, and time constraints for lunch breaks can be factors for leaving behind food (Boschini et al., 2020).

As a generalist species, the black soldier fly (BSF; *Hermetia illucens*, Diptera: Stratiomyidae) is frequently used as decomposer of organic wastes, ranging from kitchen waste (Nguyen et al., 2015) and manure (Sheppard et al., 1994) to human excreta (Banks et al., 2014). The application of BSF larvae (BSFL) can range from small-scale lab populations (Nakamura et al., 2016) and household rearing systems (Klammsteiner et al., 2020c) to large-scale industrial rearing operations (Wynants et al., 2018). Food waste is heterogenous in its composition. Elevated salt (NaCl) content in meals is often discussed because of its adverse effect on human health (e.g., high blood pressure) and can account for up to 1.2% in hot meals and sandwiches handed out in canteens (Rasmussen et al., 2010). Cho et al. (2020) concluded that BSFL are suitable to treat food waste with even higher salt concentrations, since a significant inhibition of biomass gain and pupation was only observed at concentrations >3%.

The larval gut microbiome is considered essential for efficient food conversion by BSFL. In general, results from microbiome studies on BSFL can be challenging to compare due to variations in biotic and abiotic factors during the execution of experiments (De Smet et al., 2018; Bosch et al., 2020). Recent studies suggest that, although the larval gut microbiome does not remain unaffected when exposed to various biogenic wastes, a versatile core community of bacteria may contribute to the digestion of these substrates (Wynants et al., 2018; Zhan et al., 2019; Klammsteiner et al., 2020b). Although the BSFL gut microbiome has been shown to adapt to changing diets, the overlap between the gut residing communities and communities found in the respective diets is often low (Wynants et al., 2018). Little is known about the microbial metabolism taking place in the larval guts, since past studies have mostly focused on clarifying phylogenetic dynamics in relation to larval diet. Detailed investigations of metabolic processes taking place in larvae and attempts to exploit them for biotechnological applications are sparse (Lee et al., 2018; Song et al., 2018; Zhan et al., 2019). Tools imputing metagenomes to 16S rRNA gene-based amplicon data such as PICRUSt (Langille et al., 2013) or Tax4Fun (Aßhauer et al., 2015) have been used to provide insights into functional genes. Although originally developed for a human microbiome context, the reference databases are continuously growing also for other animals and environments (Breitwieser et al., 2017; Kostanjšek et al., 2019). To comply with the widespread use of amplicon sequencing, both platforms have recently been updated to follow-up versions (Douglas et al., 2020; Wemheuer et al., 2020). Imputation methods have also become a valuable tool to gain insights in metabolic processes of insects (Chen et al., 2016) and to assess the effect of insect-based diets on livestock such as poultry (Borrelli et al., 2017). In addition, they could provide an efficient approach to screen prokaryotic communities for novel bioactive compounds and find new means for the degradation of xenobiotics as has recently been investigated in marine sponges (Steinert et al., 2019).

In this study, we determined the effect of two major organic-waste fractions occurring in canteens (food waste from leftovers and oil waste from oil separators) on BSFL growth, the bacterial gut biota, and its functional makeup. The larval diet was changed in the experiments from the chicken feed diet (CFD) to either the food (FWD) or oil waste diet (OWD) 6 days after larvae had hatched. Guts were extracted at multiple time points to determine changes in microbial-community composition and function during their development. Finally, larval growth, waste degradation, and developmental time were documented, and meaningful process indices were derived from these data. We hypothesized that, due to its high nutritional value, canteen food waste represents a convenient substrate for BSFL rearing but presumed that it would induce shifts in gut microbiome composition due to varying nutrient patterns. Moreover, we considered that diet-induced shifts in the larval gut microbiota could provide an opportunity to identify diet-specific microbial biomarkers.

## Materials and Methods

### Source of Black Soldier Fly Larvae and Colony Maintenance

Six-day old larvae were obtained from a bench-scale BSF colony at the Department of Microbiology (University of Innsbruck, Austria). Adequate and stable environmental conditions of 27°C, 60% relative humidity, and a light:dark photoperiod of 16:8 h using LED panels as described in Heussler et al. (2018) were created in a Fitotron SGC 120 (Weiss Technik, United Kingdom) climate chamber. Larvae, pupae, and adults were held in separate containers, and the density of individuals was manually controlled. An *ad libitum* amount of ground chicken feed diet (CFD; Grünes LegeKorn Premium, Unser Lagerhaus, Austria) mixed with water (40:60 w/v) was used for colony maintenance.

### Experiment Preparation and Diets

Six days after hatching, 200 manually counted larvae were each transferred to four replicate boxes per diet, adding up to a total of 2,400 larvae equally distributed over twelve separate boxes with a density of 1.2 larvae cm^–2^. The BSFL were kept under the same conditions as described for the general colony maintenance. Sterilized and dried pine humus (20 g) was added to each box as litter for humidity regulation. The diets were defined as OWD, FWD, and CFD analogous to colony maintenance as control diet. OWD and FWD were obtained from a local canteen on a single day (DMS: 47°15′50.9″ N, 11°20′37.4″ E). For OWD, the upper layers from the content of an oil separator collection container were collected and manually homogenized. Fresh canteen waste for the FWD was shredded with a Vitamix TNC^®^ electric blender (Vitamix, Olmsted Falls, OH, United States), thereby reducing the size of food residues and homogenizing its components. The diets were stored at −20°C until the start of the experiment. Feeding took place every second day for a maximum of 18 days (d_0–18_), resulting in a maximum of ten feeding events per diet. After 20 days (d_20_), the experiment was terminated. The amount of substrate to administer per larva and day was calculated using the content of organics in 100 mg CFD larva^–1^ as reference. OWD and FWD were thereby added in amounts of 70 and 170 mg larva^–1^ day^–1^, respectively. Water and organic content were determined gravimetrically by measuring the loss of mass after drying the samples in a drying oven (UF110, Memmert, Schwabach, Germany) at 105°C for 24 h and subsequent incineration in a muffle furnace (CWF 1000, Carbolite, Neuhausen, Germany) at 550°C for 5 h. Substrate-water content was adapted to the moistest substrate (FWD) by adding 70 and 100 mg of water to CFD and OWD, respectively. Thereby, all three diets contained the same amount of water and organics.

### Sample Collection and Preparation and Processing

**FIGURE 1:**
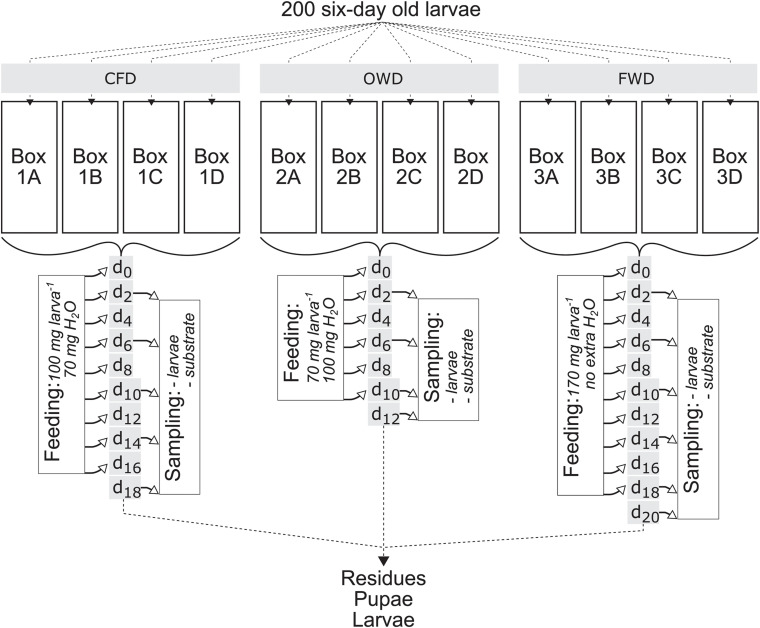
Black soldier fly (*Hermetia illucens*) larvae feeding experiment scheme displaying sampling and feeding events after the introduction of two new diets (OWD, oil waste diet; FWD, food waste diet). The 6-day old larvae were obtained from a laboratory population kept on a chicken feed diet (CFD), with the control group continuing on the same diet as before the start of the experiment. Time points d_0–20_ count the days of the experiment starting at 6 days post hatching of the larvae. Feeding events took place every other day for a maximum of 18 days.

Sampling followed the scheme illustrated in Figure 1 and samples were stored at −20°C if not immediately processed. A climate room operating at 4°C was used to slowly thaw frozen samples. One larval sample consisted of ten randomly collected larvae, of which five were used for determination of dry matter (DM) and volatile solids (VS), and another five were subjected to gut extraction. Fresh substrates and substrate residues were both physicochemically characterized prior and after the experiment, respectively. To prepare samples for the quantification of oxidizable organics [chemical oxygen demand (COD)], ammonium, total protein and reducing sugars, 10 g of sample was mixed in 25 ml diH_2_O by briefly vortexing. After incubation at room temperature for 30 min, the mixtures were centrifuged at 12,000 × *g* for 30 min and subsequently filtered (MN 615 1/4 150 mm, Macherey-Nagel, Düren, Germany). The filtrate was used for further analyses.

### Physicochemical Analyses

#### Total Solids, C/N Ratio, Volatile Solids, and Fatty Acids

Total solids were determined gravimetrically after drying the samples at 105°C for 24 h. The difference in fresh and dry mass was determined as water content. Volatile solids were inferred from the weight difference before and after incinerating the dry samples in a muffle furnace at 550°C for 5 h. Samples dried at 105°C for 24 h were finely ground using a pestle and mortar to quantify the C/N content using an elemental analyzer (TruSpec CHN Elemental Determinator, Leco, St. Joseph, MI, United States) by following the manufacturer’s protocol. For the determination of volatile fatty acids, one g per sample was mixed into 1 ml sterile water and centrifuged for 15 min at 15,000 × *g*. Vials for high performance liquid chromatography were filled with the liquid supernatant and used for analysis following Wagner et al. (2017).

#### Chemical Oxygen Demand, Ammonium, Total Protein Content, and pH

After filtration (section “Sample Collection and Preparation and Processing”), the samples were diluted in diH_2_O following the protocol enclosed in the quick test kits Nanocolor COD 1500 and Nanocolor Ammonium 50 (Macherey-Nagel, Düren, Germany), respectively, and transferred in the cuvettes. The cuvettes were incubated at 160°C for 30 min (Nanocolor Vario HC, Macherey-Nagel, Düren, Germany) and photometrically measured (Nanocolor UV/VIS, Macherey-Nagel, Düren, Germany). The Lowry protein assay following Noble and Bailey (2009) was used to determine the total protein content in sample filtrates. For the pH measurement, 10 g of substrate were mixed in 25 ml diH_2_O and briefly vortexed. After 60 min incubation at room temperature the pH was determined with a 774 pH Meter (Metrohm, Herisau, Switzerland).

### Statistical Analysis of Larval Development and Physicochemical Measurements

Growth rate, consumption index, and approximate digestibility were calculated based on Waldbauer (1968). Substrate reduction and the waste reduction index were determined as described by Jucker et al. (2020) and efficiency of conversion of the ingested and digested food was calculated as in Medrano and Gall (1976). Applied formulae are listed in Table 1. Statistical analyses were conducted in R (R Core Team, 2018). Processing of principal component analysis results and hierarchical clustering were conducted using the factoextra package (Kassambara and Mundt, 2020). All figures were created using ggplot2 (Wickham, 2016).

**TABLE 1:** Larval growth and degradation parameters based on data collected during the feeding trials (*n* = 4).

	CFD	FWD	OWD	
			
	mean ± SD	mean ± SD	mean ± SD	Formulae
Growth rate [mg d^–1^]	9.00.4^a^	8.20.8^a^	1.11.1^b^	$G\,R=\frac{{\text{L}}_{\text{end}}-{\text{L}}_{\text{initial}}}{\text{T}}$
Substrate reduction [%]	65.60.2^a^	85.30.7^b^	2.43.7^c^	$\text{SR}=\frac{I-R}{I}\times 100$
Consumption index	96.91.8^a^	156.23.9^b^	17.527.8^c^	$C\,I=\frac{E}{T\times A}$
Waste reduction index	3.70.0^a^	4.30.0^b^	0.20.3^c^	$D=\frac{I-R}{I}\,\left[c\,p\,s\,b\,r\,e\,a\,k\right]\,W\,R\,I=\frac{D}{T}\times 100$
Approximate digestibility [%]	47.70.5^a^	82.70.9^b^	–	$A\,D=\frac{E-R}{E}\times 100$
Efficiency of conversion of ingested food [%]	17.90.6^a^	7.90.9^b^	–	$E\,C\,I=\frac{B}{E}\times 100$
Efficiency of conversion of digested food [%]	37.50.9^a^	9.61.0^b^	–	$E\,C\,D=\frac{B}{E-R}\times 100$

*CFD, chicken feed diet, FWD, food waste diet, OWD, oil waste diet; SD, standard deviation. Different superscript lower-case letters indicate differences between treatments (*p* < 0.05) according to the Tukey’s HSD test. L_*initial*_, initial mean weight of larvae; L_*end*_, final mean weight of larvae; I, total feed input; R, residues; T, days; B, total larval biomass gained; A, mean fresh weight during feeding period; E, total feed ingested.*

### Harvesting of Larval Guts

After thawing, larvae were surface sterilized by briefly washing them in a Petri dish containing 70% EtOH. After the EtOH evaporated, a few mm were cut off from the anterior part of the larvae using a sterile scalpel and the gut was pulled out from this incision. From each time point × diet, 0.05 g guts were pooled from five larvae and transferred to a bead tube using sterile tweezers (Klammsteiner et al., 2020b).

### DNA Extraction and 16S rRNA Gene Amplicon Sequencing

For DNA extraction from fresh substrates, 10 g of sampling material was mixed with 25 ml deionized water. After vortexing and subsequent incubation for 30 min at room temperature, the samples were shaken for 30 min at 120 rpm (Controlled Environment Incubator Shaker, New Brunswick Scientific, United States) before filtering them through folded filters (MN 615 1/4 150 mm, Macherey-Nagel, Düren, Germany). The DNA of substrate filtrates and guts was extracted following the manual of the used kit (NucleoSpin Soil, Macherey-Nagel, Düren, Germany). Quality and quantity of extracts were assessed via agarose gel electrophoresis and spectrophotometry (NanoDrop 2000c, Thermo Fisher Scientific, Waltham, MA, United States), respectively. Biological replicates of gut samples from three different boxes per treatment were collected and prepared at each sampling time point. Amplicon sequencing including a two-step PCR library preparation using a Nextera Index Kit, purification, pooling, and demultiplexing was performed by Microsynth (Balgach, Switzerland) on a Illumina MiSeq following the 2 × 250 bp paired-end approach. The universal bacterial and archaeal primer set 515f/806r (GTGCCAGCMGCCGCGGTAA/GGACTACHVGGGTWTCT AAT) was used to amplify the V4 region on the 16S rRNA gene (Caporaso et al., 2011). The provided reads were trimmed from adaptors and primers by the sequencing provider.

### Data Analysis of Sequencing Data

Trimmed raw sequences were analyzed using mothur v.1.44.1 (Schloss et al., 2009). In the initial screening, ambiguous bases, homopolymers longer than eight and sequences >275 bases were removed. The SILVA database v.138 (Quast et al., 2013) was used as reference for alignment and classification steps. Potentially chimeric sequences were identified and subsequently removed using the vsearch algorithm. Sequences assigned to chloroplast, mitochondrial, archaeal, eukaryotic, and unknown lineages were filtered from the data. Downstream analyses were carried out in two comparative approaches: (I) binning at 97% sequence similarity and (II) identification of unique amplicon sequence variants (ASV). For data normalization, samples from both approaches were subsampled to (a) smallest sample size, (b) 15,000 sequences, and (c) 80,000 sequences. Distance matrices based on Bray-Curtis dissimilarity were calculated from the six abundance tables and pairwise comparison of matrices via Mantel test (Pearson correlation, 1000 permutations) was carried out to ensure stability of subsequent analyses (Supplementary Figure 1). The sampling effort was evaluated based on rarefaction curves (Supplementary Figure 2). Pairwise comparison of α-diversity (Shannon index; H’) in samples grouped by diet and time was conducted using analysis of variance (ANOVA), pairwise *t*-test (including Bonferroni correction), and Tukey’s honestly significant difference (HSD) test (Supplementary Table 3). Bartlett’s test was applied to test for homogeneity of variances. Non-metric multidimensional scaling (NMDS) based on Bray-Curtis dissimilarity was used as β-diversity measure on filtered gut microbiome data (minimum prevalence of four in at least 10% of samples to remove overly sparse OTUs). Overall impact of the variables diet and time on microbial communities was computed by PERMANOVA (Bray-Curtis dissimilarity, 1000 permutations). All statistical tests were conducted using vegan v.2.5.6 (Oksanen et al., 2018). Pielou’s evenness (J) was calculated as H’/log(species number). Congruent OTUs from linear discriminant analysis (LDA) effect size (Segata et al., 2011) implemented in mothur and multi-level pattern analysis as part of the R indicspecies package (De Cáceres and Legendre, 2009) were considered as indicator species.

Imputation of bacterial metabolic pathways was conducted using version 1.1.5 of the Tax4Fun2 R package and the KEGG database (Kanehisa and Goto, 2000) following the standard SOP (Wemheuer et al., 2020). Reference sequences were preclustered at 100% identity. All figures were created in R using the ggplot2 package (Wickham, 2016).

## Results

### Maturation of Larvae and Dynamics of Waste Degradation

**FIGURE 2:**
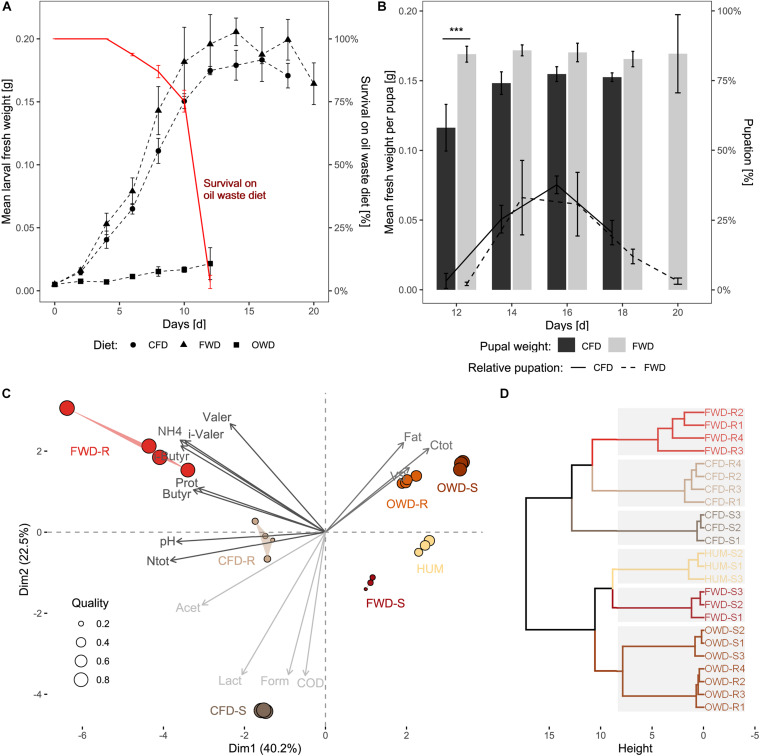
Performance of black soldier fly larvae, pupae, and their three diets. **(A)** Dotted lines represent the impact of diet on the increase of larval fresh weight (*n* = 4). The red line highlights the survival of larvae on OWD. **(B)** Bars indicate the fresh weight of pupae in relation to their time of harvest. The lines show the fraction of larvae that transitioned to pupal stage in each sampling time point. Larvae fed with OWD did not reach pupal stage and are not shown in the graph (*n* = 4). **(C)** Principal component analysis based on physicochemical properties of fresh substrates (-S) and substrate residues (-R). The three clusters of variables represented by the arrows were determined by k-means clustering and are distinguished by different shades of gray (fresh: *n* = 3; residues: *n* = 4). **(D)** Hierarchical clustering of sample replicates. Gray backgrounds represent sample clusters determined by the gap statistics method. CFD, chicken feed diet; FWD, food waste diet; OWD, oil waste diet; HUM = pine humus; Prot, proteins; Ntot, total nitrogen; Ctot, total carbon; VS, volatile solids; COD, chemical oxygen demand; (i-)Valer, (i-)valerate; (i-)Butyr, i-butyrate; Form, formate; Lact, lactate; Acet, acetate. ***p < 0.001.

The feeding experiment was observed for a maximum timespan of 20 days (d_20_) for FWD as the most prolific diet, resulting in 26-day-old larvae at the end of the experiment (Figure 1). The dietary treatments were terminated when all larvae had either pupated or died (CFD at d_18_, OWD at d_12_). Larvae on CFD and FWD both exhibited similar growth progress, with larvae offered FWD achieving a slightly higher biomass throughout the experiment (Figure 2A). However, fresh weights in the last sample were 5% lower in FWD, and biomass peaks were reached 2–6 days before the transition to pupal stage in CFD and FWD, respectively. After reaching the biomass peak at 206 ± 9 mg larva^–1^ on d_14_ and continuing their development toward pupae, biomass of FWD-fed larvae significantly decreased by 20 ± 3% until d_20_ (*p* < 0.05, ANOVA; decrease of 7 ± 5% in CFD). Drying and subsequent incineration of larvae additionally revealed similar dry matter (35–37%; of which 32–36% were volatile solids) and water contents (63–65%) in relation to fresh weight (Table 2). However, BSFL fed with OWD were strongly inhibited in their growth and further restricted by a mortality of 96 ± 2%, which ultimately led to the termination of this dietary treatment at d_12_. The lethal effect became evident after d_5_ and surviving larvae exponentially decreased until d_12_. No larva exposed to OWD transitioned to the pupal stage. The endpoint biomass per larva reared on this diet added up to 21.5 ± 1.1 mg and was thus 87% below the weight of larvae reared on CFD and FWD.

**TABLE 2:** Endpoint characterization of larval and pupal biomass (*n* = 4).

		CFD	FWD	OWD
				
		mean ± SD	mean ± SD	mean ± SD
Larvae	Fresh weight* [g]	0.171 ± 0.008^a^	0.164 ± 0.014^a^	0.022 ± 0.011^b^
	Water content* [g]	0.108 ± 0.006^a^	0.107 ± 0.012^a^	0.014 ± 0.006^b^
	Dry weight* [g]	0.063 ± 0.003^a^	0.057 ± 0.001^a^	0.008 ± 0.005^b^
	Volatile solids* [g]	0.055 ± 0.003^a^	0.054 ± 0.001^a^	0.008 ± 0.005^b^
	Mortality [%]	0^b^	0^b^	96 ± 2^a^
	Developmental time [d]	21 ± 2	22 ± 3	0
Pupae	Pupation rate [%]	87 ± 7^a^	80 ± 9^a^	0
	Fresh weight^+^ [g]	0.153 ± 0.003^a^	0.169 ± 0.023^a^	–
	Water content^+^ [g]	0.096 ± 0.001^a^	0.107 ± 0.014^a^	–
	Dry weight^+^ [g]	0.057 ± 0.001^a^	0.062 ± 0.009^a^	–
	Volatile solids^+^ [g]	0.050 ± 0.001^a^	0.060 ± 0.009^a^	–

CFD, chicken feed diet; FWD, food waste diet; OWD, oil waste diet; SD, standard deviation. Different superscript lower-case letters indicate differences between treatments (*p* < 0.05) according to the Tukey’s HSD test.

*at last sampling time point (d_20_).

*^+^after harvesting of pupae.*

With an average developmental time of 22 ± 3 days, BSFL raised on FWD developed slightly slower and with higher variability than larvae in the control group (21 ± 2 days). On CFD and FWD, first pupae appeared at d_12_ with a significantly higher initial biomass per pupa (*p* < 0.001) in FWD (Figure 2B). While pupae from FWD showed constant fresh weights of 169 ± 2 mg pupa^–1^ irrespective of the sampling event, pupae raised on CFD gradually increased from 116 ± 14 mg pupa^–1^ on d_12_ to 152 ± 2 mg pupa^–1^ in the experiment’s last sampling event on d_18_. In FWD, the last pupae were harvested on d_20_ and exhibited the largest variation in weight. Pupation in CFD reached its peak on d_16_ when biomass was highest (154 ± 5 mg pupa^–1^), resulting in around 40% of larva transitioning to pupal stage in this time point.

In both CFD and FWD, larvae showed comparable growth rates (Table 1) with approx. 8.5 mg biomass gain per day. Although substrate reduction, consumption and waste reduction indices as well as approximate digestibility were significantly higher in FWD, the efficiency of larvae to convert ingested and digested food were more than two and four times higher in the CFD control diet, respectively. Larvae raised on OWD exhibited negligible substrate reduction. The consumption and waste reduction indices indicated insignificant degradation of the diet fed.

### Physicochemical Characterization of Substrates

Principal component analysis was conducted on physicochemical data from pre-process fresh substrates used as diet (CFD-S, FWD-S, OWD-S) and in post-process substrate residues (CFD-R, FWD-R, OWD-R). The two principal components in Figure 2C explain a combined variance of 62.7% in the data. The 15 most influential parameters represented by the arrows were aggregated in three groups by k-means clustering to clarify the coordination of samples. Increased NH_4_ (20- and 30-fold in CFD-R and FWD-R, respectively) and protein (10-fold in all residues) contents strongly contributed in distinguishing pre- from post-process substrates (Supplementary Table 1). Differences between fresh and residue samples from OWD were less evident due to similar physicochemical characteristics featuring a high fat, C_tot_, and volatile solid content, therefore, both groups sharing a similar coordination. The fresh control diet CFD-S stands out from the other diets due to its elevated lactate and formate content and higher chemical oxygen demand (COD). After processing by BSFL, the residues of the control diet (CFD-R) deviated from their initial properties and shared more similarities with FWD-R, being better represented by a higher pH and N_total_ content. While FWD-S featured a comparatively scarce VFA profile (Supplementary Table 2) and a weaker fit by the principal components, the residues from this diet (FWD-R) were characterized by high protein, NH_4_, (i-)valerate, and (i-)butyrate concentrations. The VFAs together with nitrogen compounds (protein, NH_4_, N_total_) were most influential for the variation in the data. The sterilized and dried pine humus (HUM) used as neutral medium for moisture regulation during the feeding experiment contained negligible concentrations of VFAs, fats and nitrogen compounds. Relationships among groups of pre- and post-process substrates were further determined by hierarchical clustering (Figure 2D). Gap-statistics analysis (cut-off at *k* = 0.61) identified an optimal number of six clusters (from seven sample groups) represented by gray rectangles underlaying the dendrogram, summarizing the groups of fresh and residual OWD samples in one group.

### Analysis of the Substrate Microbiota

**FIGURE 3:**
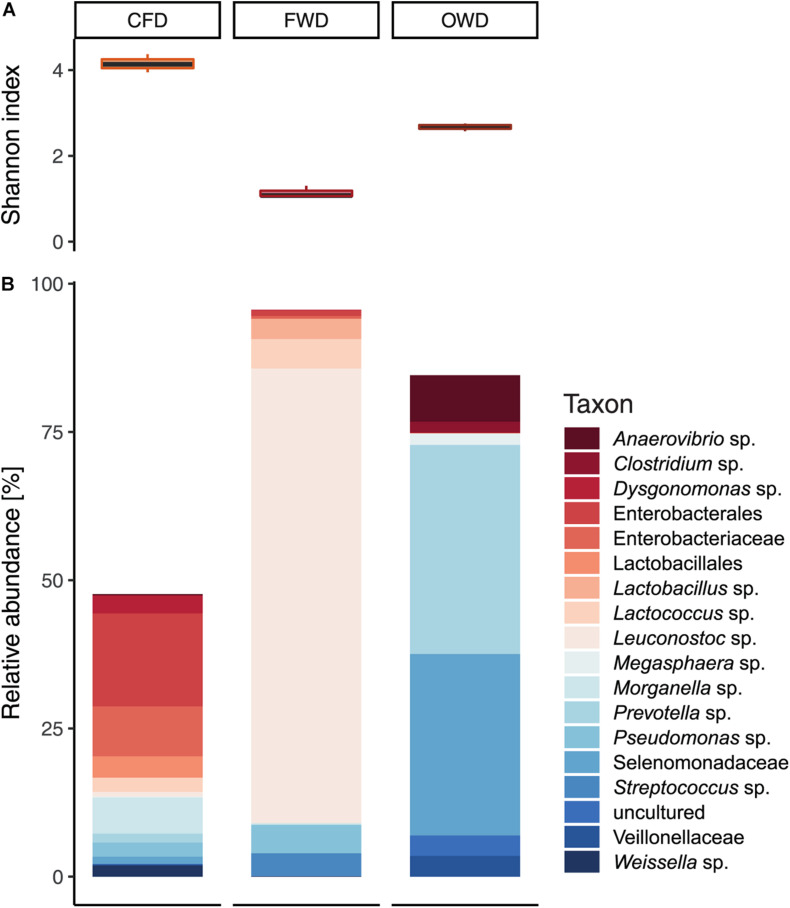
Bacterial community characteristics in chicken feed (CFD), food waste (FWD), and oil waste (OWD) administered as diet in feeding experiments with black soldier fly larvae (*n* = 3). **(A)** Shannon diversity index observed in the three diets. **(B)** Bacterial community in substrates representing taxa with a relative abundance of at least 5%.

After passing through quality control and filtering steps, a total of 7.01 × 10^5^ reads from nine fresh substrate samples (three biological replicates per substrate) were used for downstream analysis. ANOVA and PERMANOVA of fresh substrate-derived microbial communities indicated significant differences between substrates (*p* < 0.001) on α-diversity level (defined by H’; Figure 3A) and in general community composition represented by β-diversity (*p* < 0.05), respectively. Significant pairwise differences (*p* < 0.001) were further confirmed via pairwise *t*-test and Tukey’s HSD *post hoc* test (Supplementary Table 3). Firmicutes were found to be the most dominant phyla in both FWD and OWD with 92% and 57% relative abundance. CFD was dominated by Proteobacteria (44%) and contained overall lower abundances of Firmicutes (23%). Bacteroidota were most common in OWD (38%) but less abundant in FWD and CFD (28% and 14%, respectively).

With a share of 34.7 ± 3.5%, sparse OTUs with a relative abundance smaller than 1% contributed to a large part to the community in CFD compared with 8.9 ± 0.4% in OWD and 5.2 ± 0.8% in FWD. H’ further indicated a heterogeneous distribution of abundances among few OTUs in FWD (*H*’ = 1.14 ± 0.14) described by a low evenness (*J* = 0.33 ± 0.03). CFD was defined by a more even community (*J* = 0.79 ± 0.03) consisting of many less abundant OTUs (*H*’ = 4.15 ± 0.21). For matters of clarity, only genera with abundances of >5% were included in Figure 3B. On genus level, FWD was largely dominated by *Leuconostoc* (77%) of the phylum Firmicutes. In OWD (*H*’ = 2.67 ± 0.02; *J* = 0.66 ± 0.02), Firmicutes were mainly represented by unclassified Selenomonadaceae and shared their predominance (31%) with a similarly abundant *Prevotella* (36%).

### Taxonomic Exploration of the Gut Microbiota

**FIGURE 4:**
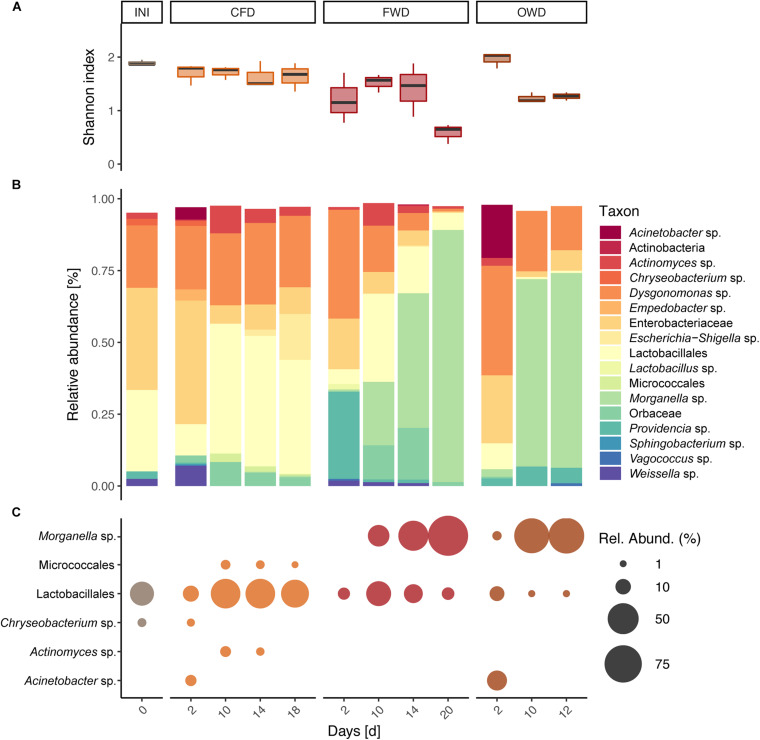
Bacterial community characteristics in guts of black soldier fly larvae reared on chicken feed (CFD), food waste (FWD), and oil waste diet (OWD). Before introducing new diets, neonate larvae were reared on CFD for 6 days (INI). Data from each time point is presented as summary of three replicates (*n* = 3). **(A)** Shannon diversity index observed in guts of BSFL. **(B)** Bacterial community composition in guts of BSFL representing taxa with a relative abundance of at least 1%. **(C)** Indicator species identified by both linear discriminant analysis of effect size and multi-level pattern analysis with a relative abundance of at least 1%.

Sequencing of the 16S rRNA V4 genetic region yielded a total of 3.50 × 10^6^ reads from 36 BSFL gut samples (three biological replicates per time × treatment) after passing through quality control and filtering steps. Mantel tests on distance matrices produced by the different clustering and subsampling approaches described in section “Data Analysis of Sequencing Data” resulted in no significant differences among the compared methods. This confirmed the overall congruency of the results irrespective of clustering method (97% similarity OTUs or unique ASVs) and subsampling size. The presented results are based on 97% similarity data subsampled to 36,878 reads (smallest sample size). The reads were clustered into 1,583 OTUs representing 491 genera within 39 phyla. Most abundant phyla across all guts were Firmicutes (30%), Proteobacteria (26%), Bacteroidota (15%), and Actinobacteriota (7%). The H’ as well as J observed in the original population of 6-day-old larvae (INI; *H*’ = 1.89 ± 0.05, *J* = 0.37 ± 0.01) remained stable throughout the CFD treatment (*H*’ = 1.65 ± 0.04, *J* = 0.35 ± 0.01) but significantly decreased in later samples obtained from the FWD treatment (*H*’ = 0.59 ± 0.18, *J* = 0.13 ± 0.04), *p* < 0.05) (Figure 4A). A similar decrease was observed in later samples from OWD-fed larvae (*H*’ = 1.25 ± 0.07, *J* = 0.25 ± 0.02).

**FIGURE 5:**
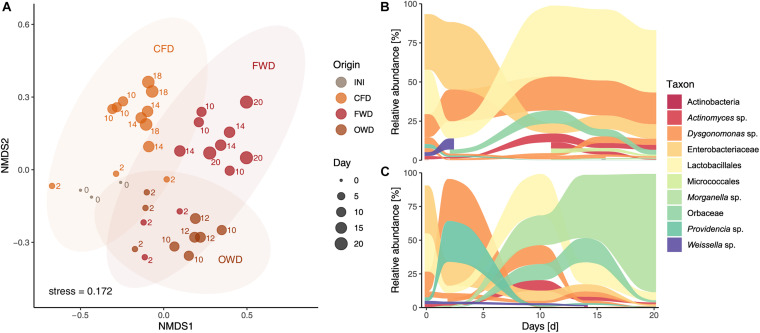
Compositional relationships and dynamics in black soldier fly guts (*n* = 3). **(A)** Non-metric multidimensional scaling of larval gut microbiome data based on Bray-Curtis dissimilarity with ellipses representing a 95% confidence interval. Data was filtered to contain OTUs with a prevalence of four in at least 10% of the samples. INI, initial population of 6-day old larvae; CFD, chicken feed diet; FWD, food waste diet; OWD, oil waste diet. Dynamics of taxa with a relative abundance >1% present in at least three sampling events in black soldier fly larvae reared on chicken feed **(B)** and food waste **(C)**.

PERMANOVA based on Bray-Curtis dissimilarity of gut microbiome data confirmed the observed differences in larval gut microbiomes (*p* < 0.001) between sampling time points and dietary treatments. Pairwise PERMANOVA with samples summarized on diet level specified significant compositional divergence from INI in the gut microbiome of FWD and OWD-fed larvae but showed stability in the CFD control group (Figure 4B and Supplementary Table 3). While *Dysgonomonas* (38%) and *Providencia* (30%) were initially highly prevalent in FWD, over time they were displaced by *Morganella* (from 1% in d_2_ to 88% in d_20_; Figure 4B). A comparable development was observed in OWD, which contributed to an increasing similarity to FWD-fed larvae. NMDS analysis of the larval gut microbiome data (stress < 0.2) further explained spatial relationships between diets and highlighted the compositional overlap found in FWD and OWD-fed larvae (Figure 5A). Linear discriminant analysis of effect size found 30 OTUs to be explanatory for differences between groups, while 49 OTUs were identified to be strongly associated with the respective dietary treatment by multi-level pattern analysis. A congruent set of 17 OTUs recognized by both methods was defined as indicator species and assigned to 15 distinct genera (genera with relative abundance >1% in Figure 4C). Representatives of the order of Lactobacillales were found to be characteristic in guts of all treatments, but could not be assigned to a described genus. *Morganella* contributed with high abundances to the differentiation of mid to late stages of FWD- and OWD-fed larvae from the control group. However, CFD-fed larvae contained specific differentially abundant bacterial groups depending on their stage of growth. *Chryseobacterium* was found to be distinctive for early stages, while *Actinomyces* and Micrococcales were mostly found in mid and late stages. Moreover, the later samples spread further away from the initial community (INI) that consisted of predominantly Enterobacteriaceae (36%), Lactobacillales (29%), and *Dysgonomonas* (21%). Although similar in larval development time and growth rate, CFD and FWD-fed larvae exhibited different dynamics of the most abundant taxa in their guts. Persistent genera with a relative abundance >1% and present in at least three sampling events are shown in Figures 5B,C for CFD and FWD-fed larvae, respectively.

### Imputation of Main Metabolic Pathways of the Gut Microbiota

**FIGURE 6:**
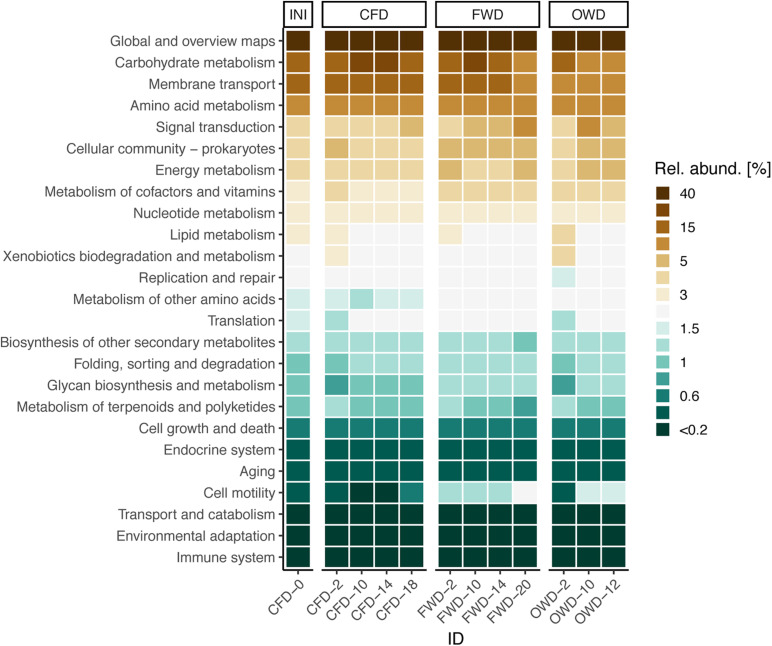
Most abundant functional pathways in the gut microbiota of black soldier fly larvae before introduction of dietary treatments (INI) and after introduction of food waste (FWD) and oil waste (OWD) (*n* = 3). The control group was further fed with chicken feed (CFD). Pathways were obtained from Tax4Fun2 analysis and summarized on level 2 of the BRITE hierarchy based on KEGG orthology.

Approaches making use of metagenomic imputation as introduced by the R packages Tax4Fun2 were used to infer metabolic pathways from 16S rRNA gene-based amplicon data. On average, 70 ± 18% of the obtained sequences could be assigned to the reference database and used for the prediction of pathways. PERMANOVA analysis indicated significant time- and diet-dependent differences (*p* < 0.001) in metabolic profiles (Supplementary Table 3). Pairwise comparison of data grouped on diet-level via pairwise PERMANOVA revealed that both OWD and FWD differ from the CFD control group, but only OWD diverged from the metabolic pathways found in larval gut communities of INI. In contrast to the deviating development of FWD from INI on a phylogenetic level, this difference was not observed in a metabolic context. In general, the distinction between treatments on the level of metabolic pathways was much blurrier than on a taxonomic level (Figures 4B, 6).

## Discussion

Two major fractions of canteen waste – FWD and OWD – were compared with a commonly used CFD. Impact on the growth, the gut microbiome profiles, and the divergence from the initial 6-day old population of BSFL were determined.

### Canteen Food Waste as an Easily Degradable and Growth-Promoting Substrate for BSFL

Food waste from canteens is highly heterogenous and mainly consist of staples (potatoes, rice, pasta), vegetables/fruits, and grain products with smaller amounts of meat and fish wastes (Silvennoinen et al., 2015). Our FWD derived from one batch of mixed organic wastes that accumulated on a single day in a local canteen. We found that larvae fed FWD took a highly similar course in biomass gain and development as the CFD-fed control group (Figures 2A,B). However, endpoint biomass of FWD-fed larvae were 5% below the average measured in the control. These results are congruent with previously published records of larvae raised on chicken feed (on average, 158 ± 20 mg larva^–1^; Barragán-Fonseca et al., 2017). While CFD-fed larvae did not experience a considerable decrease in biomass once they had reached their peak weight, FWD-fed larvae approaching (pre-)pupal stage consistently lost mass. This is crucial in calculating cost efficiency and designing processes in the large-scale application of BSFL (Salomone et al., 2017). A biomass peak significantly higher a few days prior to the transition to pupal stage suggests not to use the self-harvesting ability of larvae in an industrial setting. Instead, a batch system with scheduled mechanical or manual harvesting cycles taking place during the fifth instar before onset of migration could represent a more efficient option. Although greenhouse gas emission and overall environmental footprint of treating organic wastes with BSFL were already low compared with traditional composting (Mertenat et al., 2019), timing the harvest of larvae could boost the efficiency of operating resources. Improved biomass output and more stable frequency of rearing cycles would be the consequences. The advantages of harvest before the last instar became also evident when larvae were used for the production of biodiesel, since they contained a higher fraction of lipids and less chitin (Wong et al., 2019).

During the degradation process, FWD was highly modified by larval and microbial activity. This is indicated by high concentrations of VFAs such as valerate and butyrate, but also higher NH_4_ contents (Figure 2C). The accumulation of nitrogen compounds is of interest for the commercialization of residues as soil amendment (Klammsteiner et al., 2020a). Residues from BSFL-processed household organic wastes showed similar C_tot_ and N_tot_ ratios to the FWD-R produced in our study and have been identified as an effective nitrogen source for plant growth (Kawasaki et al., 2020). Moreover, the generally high chitin content in residues produced by insects can improve the defense against phytopathogens by triggering plant-based immune responses (Sharp, 2013).

When food waste remains untreated, microbial spoilage dominated by lactic acid bacteria can cause the pH to rapidly drop to approx. 4.3 within 7 h (Aichinger et al., 2015; Wu et al., 2018). A similarly low initial pH of 4.5 was observed in the food waste fed to the larvae in our study (Supplementary Table 1), before shifting to a more neutral value in post-process residues (Figure 2C). This is particularly important from a hygiene perspective, as these acidic conditions can inhibit bacterial pathogens (Wu et al., 2018). In addition, BSFL have been shown to exhibit antimicrobial activity against multiple potentially human pathogenic bacteria by excreting antimicrobial compounds into their environment (Erickson et al., 2004; Liu et al., 2008; Choi et al., 2012).

Larvae fed with FWD showed significantly enhanced substrate reduction, better approximate digestibility of the substrate, and a higher consumption index compared with CFD (Table 1). However, a presumably lower nutrient availability in this diet required the ingestion of larger amounts to reach the same larval biomass as was observed in CFD (Table 2). This is emphasized by the comparably low efficiency of FWD-fed larvae to convert ingested and digested food to their own biomass. The chicken feed commonly used to maintain BSF lab populations was developed as a balanced, energy-rich diet for laying hens and optimized amounts for BSFL rearing have been investigated by Diener et al. (2009). The overall benefit created by using waste-derived substrates, however, can make up for the lower biomass yield and slower development. It is still not known whether the larvae adapt to a specific diet over time and how many generations are necessary to establish metabolic stability. Nutrition-induced epigenetic inheritance from previous generations has been found to influence offspring phenotypes in other Dipterans such as *Drosophila melanogaster* (Wang et al., 2017). The potential distortion and its extent during impact assessment of newly introduced diets on BSFL growth and microbe-host relationships have yet to be quantified.

### Oil-Separator Waste Is Strongly Reducing BSFL Development and Survival

The OWD treatment mainly derived from fats, oils, and grease (FOG) trapped as supernatant in the collection container of an oil separator. In addition to the lipid fraction, it also contained a smaller and heterogenous fraction of food residues from the cooking process. The fatty components of the OWD represent a more problematic fraction of the canteen waste since FOGs generally require more refined treatment methods (Husain et al., 2014). Supplying them to anaerobic digestion is a frequently chosen biotreatment (Long et al., 2012), but also composting of FOG compounds provided promising results (Lemus and Lau, 2002).

In our trial, the OWD changed its consistency within a few days and became more viscous, making it harder for the larvae to move. However, physicochemical properties and volatile fatty acid profiles remained largely unchanged (Figures 2C,D and Supplementary Tables 1, 2). Solidification and deposition of FOGs are a widespread problem in sewer systems transporting wastewater from food service establishments (He et al., 2013). These processes are most likely driven by high concentrations of saturated fats and calcium, while moisture content is secondary (Keener et al., 2009). Due to the slow growth rate and negligible substrate reduction, the degradation parameters did not provide any meaningful information.

A combination of inhibited mobility and lack of easily degradable nutrients may have led to the starvation of the larvae. Moreover, the physical blocking of the larvae’s spiracles by the oil could have led to their suffocation (Taverner, 2002). After approximately 7 days, the extent of this effect became visible by an exponentially increasing mortality (Figure 2A). No notable ingestion and digestion of the substrate could be assessed by the consumption and waste reduction index (Table 1). There have not been in-depth investigations on BSF lipid metabolism yet, but a recent study showed that BSFL do not only bioaccumulate fatty acids from their diet but are also able to synthesize fatty acids themselves (Hoc et al., 2020). Future studies with diets supplemented with defined combinations of FOGs could determine limits of BSFL fat tolerance in organic wastes to enable FOG utilization in BSFL feedstuff.

### Food Waste Diet Shapes the BSFL Gut Microbiome

Chicken feed diet-fed larvae retained a stable bacterial diversity throughout the experiment and did not diverge microbiome-wise from the initial population of 6-day old larvae raised on the same diet (Figures 4A,B). These observations are in line with Cifuentes et al. (2020), who found that a chicken-feed-based diet contributes to an overall stable gut microbiome during larval development. The introduction of FWD and OWD treatments had a decisive impact on the development of the BSFL gut microbiome. Over time, the initially abundant Lactobacillales, Enterobacteriaceae, and *Dysgonomonas* were displaced by mostly *Morganella* in both diets. This compositional change further led to a decrease in α-diversity by crowding-out other groups and thereby. Even though larval growth (Table 2) and substrate degradation parameters (Table 1) reported negligible conversion of OWD, the gut microbiomes of these larvae developed similarly to FWD-fed larvae (Figure 4B). However, in contrast to Raimondi et al. (2020), we could not observe a meaningful increase of gut microbiome complexity over time.

As recently stated by Raimondi et al. (2020), Proteobacteria, especially *Providencia*, dominated guts of vegetable-fed larvae irrespective of the rearing temperature used in their study. In our trial, however, *Providencia* was mostly found in early larval stages and in low abundances throughout the development of OWD-fed larvae. This discrepancy is likely caused by the nutritional differences between their standard vegetable diet and the FWD and OWD used in our study. Larval ingestion of corn flour, wheat bran, and alfalfa flour contained in the vegetable diet might favor the prevalence of *Providencia* (Raimondi et al., 2020).

Furthermore, the Gram-negative and motile genus of *Morganella*, predominant in FWD and OWD-fed larvae, is known to share several biochemical aptitudes such as the (oxidative) deamination of certain amino acids with *Providencia* (Manos and Belas, 2006). It has previously been found to play a significant role in the BSFL gut microbiome due to its prevalence across various feeding schemes and larval developmental stages (Wynants et al., 2018; Cifuentes et al., 2020; Klammsteiner et al., 2020b; Liu et al., 2020; Raimondi et al., 2020). In our study, *Morganella* strongly contributed as indicator species to the distinction of FWD- and OWD-shaped gut microbiomes from the control group (Figure 4C). It is an environmentally widespread genus of the Enterobacterales so far only consisting of the species *Morganella morganii* and is often found as commensal in human guts (Manos and Belas, 2006). Due to its potential to cause severe infections, it has been cited as a relevant pathogenic microorganism associated with insects grown for feed and food (Schlüter et al., 2017; Raimondi et al., 2020). As opposed to rearing BSFL raised on CFD, food waste favored the enrichment of *Morganella* in larval guts. In fresh FWD and OWD substrates, *Morganella* was only present in comparatively low relative abundances of 0.1–0.3% while fresh CFD contained 6% but did not promote its accumulation in larvae. Additionally, extensive gut colonization by other bacteria was inhibited as has previously been observed in carrion degrading burying beetles: while endogenous *M. morganii* not only contributed to the stabilization of the beetle gut microbiome, it also helped to outcompete rival bacterial communities and prevent colonization by potentially entomopathogenic bacteria (Duarte et al., 2018; Wang and Rozen, 2018). High abundances of this species were also reported for other Diptera such as wild populations of the Mediterranean fruit fly *Ceratitis capitata* (De Cock et al., 2019). On the contrary, for mass-reared Mexican fruit flies (*Anastrepha ludens*), inoculating the substrate with only 105 CFUs ml^–1^ of *M. morganii* led to 100% mortality in larvae while lower concentrations reduced emergence and flight ability of adults (Salas et al., 2017). Although *Morganella* did not negatively alter BSFL performance in our trial, the role of other entomopathogens as a risk factor in industrial *H. illucens* production remains to be investigated. Immunological responses of larvae in form of antimicrobial peptides have been addressed (Vogel et al., 2018). However, microorganisms (bacteria, fungi, and viruses) potentially endangering the uprising BSF industry – this includes fly populations as well as workplace safety – are due to be characterized (Joosten et al., 2020).

Since previously reported studies rarely match in rearing conditions, protocols, and locations, detailed comparisons are strongly limited (De Smet et al., 2018). Shifts, and thereby adaptations, of gut microbial communities to new diets might be primarily driven by the functional needs involved in degrading the available substrate. By clarifying whether in BSFL metabolic competence is preferred over enrichment of ingested exogenous microbes, the accumulation dynamics of specific phylogenetic groups in the larval gut could further be explained. In this context, investigating also the modification of the inherent digestive enzymatic toolkit and gut cell morphology in relation to diet uptake constitutes a crucial approach to gain a comprehensive picture of larval metabolism (Bruno et al., 2019; Bonelli et al., 2020). Nevertheless, we think that especially the pursuit to convert organic wastes known to carry a high bioburden into larval biomass should provide further motivation to find appropriate pre-treatment methods for substrates.

### Indicator Species as Traits for Dietary Adaptation of Microbial Communities in the Gut

Besides *Morganella*, Lactobacillales pervaded guts from all treatments (Figure 4C). They have been identified to be a common driving factor for the divergence of dietary treatment groups. Although the OTUs representative of this group could not be classified at the genus level, a selection of Lactobacillales taking place based on the ingested diet is probable. This order also includes lactic acid bacteria such as *Lactobacillus*, *Leuconostoc*, and *Lactococcus*. In extensively studied honeybees and fruit flies, probiotic lactic acid bacteria were found to improve pesticide resistance and gastrointestinal pathogen control (Trinder et al., 2015; Daisley et al., 2017). Moreover, inoculation of organic wastes with Lactobacilli was shown to yield a higher biomass output and a better nutritional spectrum in BSFL compared with artificial feed-amended wastes (Somroo et al., 2019). Depending on their distribution at a higher phylogenetic resolution (e.g., species level), Lactobacillales identified as indicator species could act as candidate biomarker for larva-substrate interactions. Wu et al. (2018) found that lactic acid bacteria, and in particular Lactobacillales, strongly prevail during the microbial degradation of food waste and shape its microbiome by changing physicochemical parameters.

In the CFD-fed control group, a temporal succession of low-abundant indicator species was observed. *Chryseobacterium* defined the guts of early CFD-fed larvae and was previously linked to the digestion of high-fiber diets in guts of American cockroaches (Dugas et al., 2001). Two Actinobacteria represented by *Actinomyces* and Micrococcales, were found to be specific for mid to late-stage larvae. These findings are consistent with observations made by Raimondi et al. (2020), who identified Micrococcales (in particular *Brevibacterium*) as biomarker in prepupae. In a previous study, we identified *Actinomyces* as a main member of the gut core microbiome in larvae raised on low-bioburden diets such as commercial chicken feed (Klammsteiner et al., 2020b).

### Main Metabolic Features Are Not Primarily Defined by Community Composition

Deducing metabolic pathways from short reads and microbial abundances comes with limitations but can maximize the informative value of marker-gene data. Especially environmental samples have more shortcomings compared with human-derived samples. Poorer availability of closely related reference genomes and the reliance on comparatively short sequence fragments that are often highly similar among distinct bacterial families have to be kept in mind (Knight et al., 2018). However, deducing links between taxonomy and function from 16S rRNA gene-based amplicon data can also be a preferable method to shotgun metagenomic sequencing, when, e.g., the host genome DNA strongly interferes with the microbiome signal at sequencing step (Langille, 2018). This could pose a problem when using metagenomic sequencing to investigate gut microbial communities in BSFL, as samples still contain host derived tissues.

Because of the known limitations, we decided to conduct our comparative analysis on the second level of the KEGG hierarchy instead of focusing on distinct pathways and/or metabolic functions. Although the administered diets induced clear shifts in the composition of microbial communities, the differences in the main metabolic pathways between dietary treatments were not as clear (Figure 6). A similar conclusion was reached by Zhan et al. (2019) on the larval transcriptome level: to help with the digestion of food waste as well as poultry, swine, and dairy manure, larvae use a common genetic toolkit instead of expressing diet-specific genes. These basic findings are directly in line with findings of The Human Microbiome Project Consortium (2012). A comprehensive study of the human microbiome led to the conclusion that metabolic pathways are mostly stable despite larger variations in microbial community structure. Pathways taking care of general and specific metabolic functions (carbohydrate, amino acid, energy, cofactor, and vitamin metabolism), membrane transport, and cell communication were amongst the highest abundant pathways in BSFL gut microbiomes.

So far, the investigation of metabolic functions of the BSFL gut microbiome has received little attention. Integrating metagenomic and metatranscriptomic analyses in future studies as expansion of imputed metagenomics could help to find a consensus in describing the functional framework in which BSFL convert organic wastes to biomass.

## Conclusion

Decentralized collection and processing centers in hubs of social activity could represent a feasible way to valorize food waste. By premixing the wastes collected from various sources, a more average and stable larval feedstuff composition could be achieved. The high content of fat, oil, and grease in the oil waste fraction of canteen wastes was found to be lethal for BSFL, but the conversion of the food waste fraction proceeded efficiently. Diet-induced shifts in gut microbiota were observed throughout the larval development. In contrast to the diet adaption on a phylogenetic level, general functional competences described as metabolic pathways resulted in similar patterns irrespective of the diet. This indicates that metabolic competence is a strong filter in the selection of larval gut colonizers, meaning that different communities can do the same job. Yet, many aspects of BSFL metabolism, especially fatty-acid digestion and accumulation as well as substrate-larva-microbiome interdependencies are still largely undescribed. Special attention should also be given to the assessment of risk factors such as entomopathogens and enrichment of potentially human-pathogenic microorganisms introduced by contaminated wastes.

## Data Availability Statement

The datasets presented in this study can be found in online repositories. The names of the repository/repositories and accession number(s) can be found below: https://www.ebi.ac.uk/ena, PRJEB39545.

## Author Contributions

TK performed statistical and bioinformatical data analysis, created the figures, and wrote the manuscript. AW designed and supervised the experiment. TB carried out sample collection and preparation, physicochemical analyses, and extraction of DNA. CH maintained the BSF colony, provided larvae for the experiment, and assisted during the experimental analyses. BS assisted with bioinformatical analysis and writing. FS, BS-S, and HI contributed equally to the study by supervising the planning, execution, and analysis of the study and provided scientific input during writing. All authors contributed important intellectual content and approved the final version of manuscript for publication.

## Conflict of Interest

The authors declare that the research was conducted in the absence of any commercial or financial relationships that could be construed as a potential conflict of interest.

## Abbreviations

ANOVA: analysis of variance
BSF: black soldier fly
BSFL: black soldier fly larvae
CFD: chicken feed diet
COD: chemical oxygen demand
DM: dry matter
FOG: fats and grease
FWD: food waste diet
NMDS: non-metric multidimensional scaling
OTU: operational taxonomic unit
OWD: oil waste diet
VFA: volatile fatty acids
VS: volatile solids.
