# Supplementary material for: Impact of Processed Food (Canteen and Oil Wastes) on the Development of Black Soldier Fly (Hermetia illucens) Larvae and Their Gut Microbiome Functions
Source: Front Microbiol. 2021 Jan 21;12:619112. doi: 10.3389/fmicb.2021.619112 (PMC7858275; doi:10.3389/fmicb.2021.619112)
Supplement: Supplementary file 1 [file Data_Sheet_1.PDF]

Supplementary Material

1 Supplementary Figures and Tables

1.1 Supplementary Figures

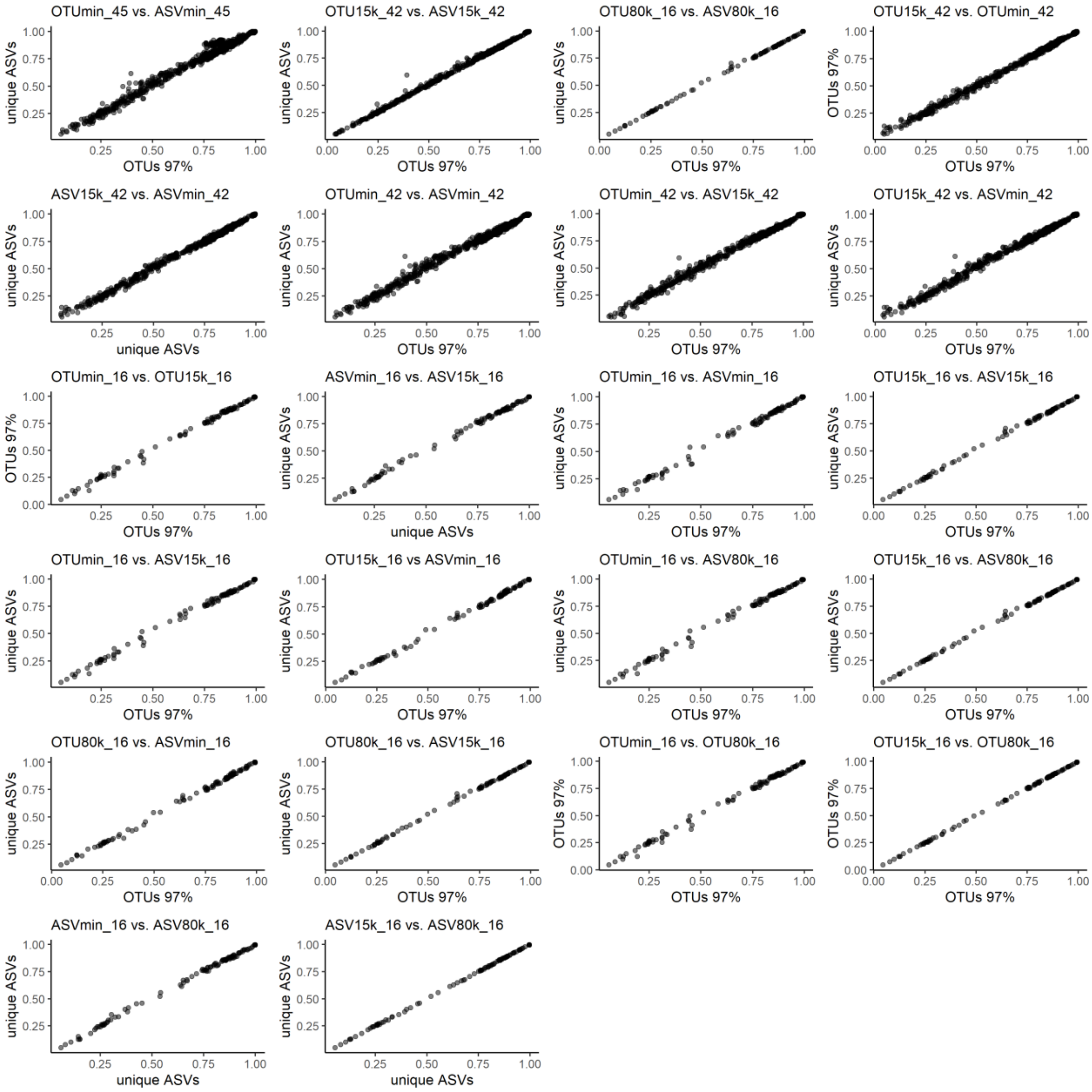

Supplementary figure 1. Correlation plots based on Bray-Curtis dissimilarity calculated from abundance tables to assess stability of the sequence data. The OTU abundance tables were produced by 22 combinations of sequence clustering approaches and subsample parameters. (97% sequence similarity vs. unique amplicon sequence variants and subsampling to 80,000 sequences, 15,000 sequences, and smallest sample size).

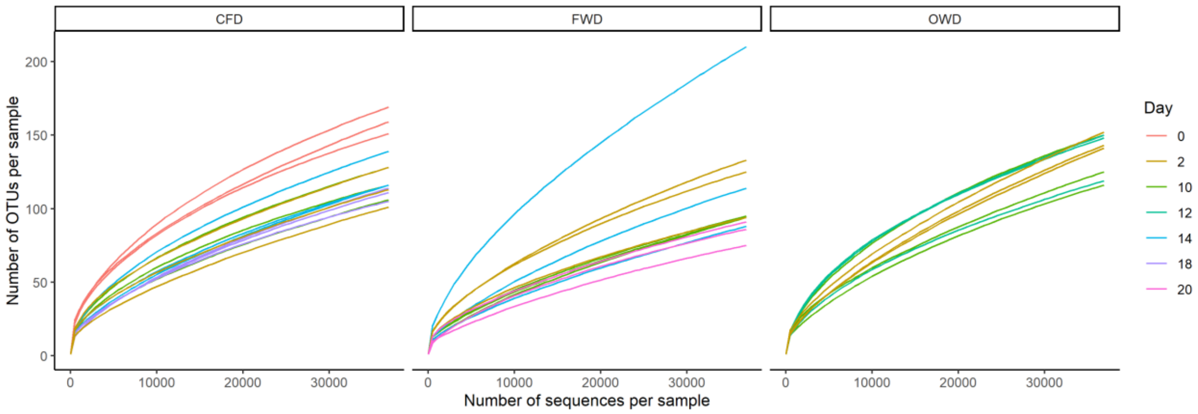

Supplementary figure 2. Rarefaction curves of sequence data clustered at 97% similarity and subsampled to the smallest sample size of 36,878 reads.

1.2 Supplementary Tables

Supplementary table 1. Physicochemical measurements of pre- and post-process substrates used as diet (n = 4).

	pH	Dry matter [%]	Volatile solids [%]	COD [mg l^{-1} O_2]	Fat [%]	C_{total} [%]	N_{total} [%]	Protein [mg ml^{-1}]	NH_4 [mg ml^{-1} NH_4-N]	Reducing sugars [g l^{-1}]
CFD-S	5.92 ± 0.00	90.13 ± 0.20	85.26 ± 0.56	18027 ± 384	3.6 ± 0.2	41.0 ± 0.6	2.95 ± 0.07	3.59 ± 0.14	9.2 ± 1.2	3.78 ± 0.85
CFD-R	6.22 ± 0.04	86.77 ± 0.68	80.39 ± 0.52	3537 ± 3372	4.1 ± 0.9	40.1 ± 0.7	3.36 ± 0.05	35.99 ± 5.77	214.8 ± 42.3	< 0.50
FWD-S	4.48 ± 0.00	19.35 ± 0.38	94.22 ± 0.20	8920 ± 1138	5.3 ± 0.4	46.7 ± 0.4	2.76 ± 0.14	2.53 ± 0.23	16.9 ± 1.1	7.69 ± 1.45
FWD-R	6.31 ± 0.04	80.30 ± 1.30	88.05 ± 0.70	2653 ± 174	9.7 ± 0.6	44.3 ± 1.6	4.01 ± 0.22	26.83 ± 7.06	557.9 ± 134.3	6.37 ± 0.84
OWD-S	4.05 ± 0.01	54.13 ± 1.78	99.79 ± 0.03	3657 ± 15	29.7 ± 5.0	71.0 ± 0.2	0.70 ± 0.08	0.30 ± 0.02	21.7 ± 0.6	< 0.50
OWD-R	4.99 ± 0.04	75.84 ± 2.56	97.53 ± 0.30	4797 ± 620	39.1 ± 3.5	66.8 ± 1.1	0.67 ± 0.06	3.58 ± 1.31	78.4 ± 16.8	< 0.50
Humus	3.31 ± 0.01	61.45 ± 0.93	81.73 ± 4.77	940 ± 25	0.3 ± 0.1	39.8 ± 0.5	0.43 ± 0.02	1.28 ± 0.07	< 1.0	< 0.50

COD, chemical oxygen demand; C_{total}, total carbon; N_{total}, total nitrogen; CFD, chicken feed diet; FWD, food waste diet; OWD, oil waste diet; -S, fresh substrates before processing; -R, substrate residues after processing; Humus, sterilized, dried pine humus as neutral litter for humidity regulation.

Supplementary table 2. Volatile fatty acid (VFA; g ml^{-1}) profiles of pre- and post-process substrates used as diet (n = 4).

	Formate	Acetate	Propionate	Butyrate	Lactate	i-Butyrate	i-Valerate	Valerate
CFD-S	4.18 ± 0.00	56.97 ± 0.00	1.11 ± 0.00	3.67 ± 0.00	35.68 ± 0.00	ND	0.53 ± 0.00	ND
CFD-R	ND	9.80 ± 1.19	5.59 ± 1.38	1.26 ± 0.17	15.46 ± 1.26	36.91 ± 3.35	8.84 ± 0.72	ND
FWD-S	ND	32.06 ± 0.00	16.06 ± 0.00	2.51 ± 0.00	18.83 ± 0.00	ND	ND	1.65 ± 0.00
FWD-R	ND	45.82 ± 7.43	4.44 ± 7.46	7.79 ± 3.33	9.40 ± 2.03	77.66 ± 20.18	28.30 ± 7.98	10.90 ± 2.19
OWD-S	ND	7.42 ± 0.00	29.73 ± 0.00	2.27 ± 0.00	0.12 ± 0.00	2.73 ± 0.00	1.49 ± 0.00	4.86 ± 0.00
OWD-R	ND	2.10 ± 0.67	0.18 ± 0.08	1.29 ± 0.23	0.04 ± 0.03	7.07 ± 1.11	3.53 ± 0.63	1.21 ± 0.16
Humus	ND	ND	0.78 ± 0.00	0.10 ± 0.00	ND	ND	ND	ND

ND, not detected; CFD, chicken feed diet; FWD, food waste diet; OWD, oil waste diet; -S, fresh substrates before processing; -R, substrate residues after processing

Supplementary table 3. Statistical analysis of substrate and gut microbial community data. Depending on the analysis, samples were grouped either based on ID (corresponds to merged triplicates of each sampling time point and treatment) or substrate level (samples from each treatment were merged). CFD, chicken feed diet; FWD, food waste diet; OWD, oil waste diet; Df, degrees of freedom; SumofSqs, sum of squares; MeanSq, mean sum of squares; diff, difference in observed means; lwr, lower end point of interval; upr, upper end point of interval; p adj, adjusted p-value.

I. Substrate samples

Bartlett test of homogeneity of variances		
Bartlett's K-squared	Df	p-value
1.1317	2	0.5679

ANOVA (beta diversity data based on Shannon index)					
	Df	SumofSqs	MeanSq	F-value	Pr(>F)
(Intercept)	1	63.45	63.45	2684.1	3.47e-09***
ID	2	13.58	6.79	287.4	1.10e-06***
Residuals	6	0.14	0.02		

Tabulated F-value: 19.32953

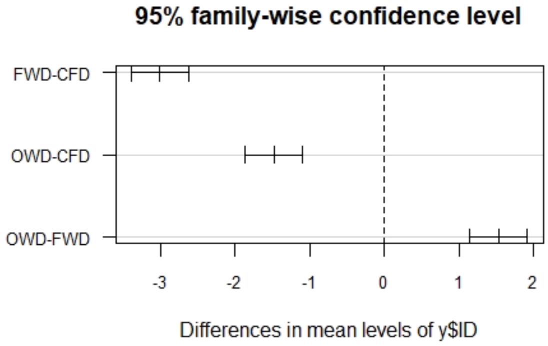

Pairwise t-test (Bonferroni correction)		
	CFD	FWD
FWD	1.0e-06	-
OWD	6.7e-05	5.6e-05

TukeyHSD				
	diff	lwr	upr	p adj
FWD-CFD	-3.0092	-3.3944	-2.6241	1.00e-06
OWD-CFD	-1.4801	-1.8653	-1.0950	5.55e-05
OWD-FWD	1.5291	1.1440	1.9143	4.59e-05

Permutational multivariate ANOVA (PERMANOVA) of substrate microbiome data (Bray-Curtis)						
	Df	SumsOfSqs	MeanSqs	F.Model	R^2	Pr(>F)
Substrate	2	2.64557	1.32278	46.024	0.9388	0.002997**
Residuals	6	0.17245	0.02874		0.0612	
Total	8	2.81802			1.0000	

*Signif. codes: 0 '***' 0.001 '**' 0.01 '*' 0.05 '.' 0.1 ' ' 1; number of permutations: 1000*

II. Gut samples

Bartlett test of homogeneity of variances		
Bartlett's K-squared	Df	p-value
15.598	11	0.1567

ANOVA of gut microbiome beta diversity data based on Shannon index					
	Df	SumofSqs	MeanSq	F-value	Pr(>F)
(Intercept)	1	78.87	78.87	1250.284	< 2e-16***
ID	11	4.62	0.42	6.652	5.52e-05***
Residuals	24	1.51	0.06		

Tabulated F-Value: 2.608974

Pairwise t-test (Bonferroni correction)											
	CFD-0	CFD-10	CFD-14	CFD-18	CFD-2	FWD-10	FWD-14	FWD-2	FWD-20	OWD-10	OWD-12
CFD-10	1.0000	-	-	-	-	-	-	-	-	-	-
CFD-14	1.0000	1.0000	-	-	-	-	-	-	-	-	-
CFD-18	1.0000	1.0000	1.0000	-	-	-	-	-	-	-	-
CFD-2	1.0000	1.0000	1.0000	1.0000	-	-	-	-	-	-	-
FWD-10	1.0000	1.0000	1.0000	1.0000	1.0000	-	-	-	-	-	-
FWD-14	1.0000	1.0000	1.0000	1.0000	1.0000	1.0000	-	-	-	-	-
FWD-2	0.1875	1.0000	1.0000	1.0000	1.0000	1.0000	1.0000	-	-	-	-
FWD-20	0.0001	0.0008	0.0020	0.0019	0.0010	0.0081	0.0325	0.3689	-	-	-
OWD-10	0.2421	1.0000	1.0000	1.0000	1.0000	1.0000	1.0000	1.0000	0.2869	-	-
OWD-12	0.3715	1.0000	1.0000	1.0000	1.0000	1.0000	1.0000	1.0000	0.1862	1.0000	-
OWD-2	1.0000	1.0000	1.0000	1.0000	1.0000	1.0000	0.8819	0.0831	0.0000	0.1078	0.1673

Permutational multivariate ANOVA (PERMANOVA) of gut microbiome data (Bray-Curtis)						
	Df	SumsOfSqs	MeanSqs	F.Model	R^2	Pr(>F)
ID	11	6.2307	0.56643	6.6965	0.75425	0.000999***
Residuals	24	2.0301	0.08459		0.24575	
Total	35	8.2608			1.00000	

*Signif. codes: 0 '***' 0.001 '**' 0.01 '*' 0.05 '.' 0.1 ' ' 1; number of permutations: 1000*

Pairwise PERMANOVA on gut microbiome data (Bray-Curtis dissimilarity; samples grouped on substrate level)			
	CFD	FWD	INI
FWD	0.012	-	-
INI	0.719	0.047	-
OWD	0.006	1.000	0.024

Number of permutations: 1000; p-value adjustment: Bonferroni

PERMANOVA on metabolic pathways obtained from Tax4Fun2 analysis on gut microbial communities (Bray-Curtis)						
	Df	SumsOfSqs	MeanSqs	F.Model	R^2	Pr(>F)
ID	11	0.11427	0.0103877	9.7807	0.81761	0.000999***
Residuals	24	0.02549	0.0010621		0.18239	
Total	35	0.13975			1.00000	

*Signif. codes: 0 '***' 0.001 '**' 0.01 '*' 0.05 '.' 0.1 ' ' 1; number of permutations: 1000*

Pairwise PERMANOVA on metabolic pathways obtained from Tax4Fun2 analysis on gut microbial communities (Bray-Curtis dissimilarity; samples grouped on substrate level)			
	CFD	FWD	INI
FWD	0.012	-	-
INI	1.000	0.503	-
OWD	0.006	0.545	0.042

Number of permutations: 1000; p-value adjustment: Bonferroni

TukeyHSD post-hoc test of gut microbiome data

$ID	diff	lwr	upr	p adj
CFD-10-CFD-0	-0.1755	-0.9149	0.5640	0.9990
CFD-14-CFD-0	-0.2531	-0.9925	0.4864	0.9801
CFD-18-CFD-0	-0.2525	-0.9919	0.4870	0.9805
CFD-2-CFD-0	-0.1939	-0.9333	0.5455	0.9976
FWD-10-CFD-0	-0.3676	-1.1070	0.3718	0.8068
FWD-14-CFD-0	-0.4802	-1.2196	0.2592	0.4768
FWD-2-CFD-0	-0.6816	-1.4211	0.0578	0.0902
FWD-20-CFD-0	-1.3059	-2.0453	-0.5665	0.0001
OWD-10-CFD-0	-0.6601	-1.3996	0.0793	0.1113
OWD-12-CFD-0	-0.6236	-1.3631	0.1158	0.1566
OWD-2-CFD-0	0.0676	-0.6718	0.8070	1.0000
CFD-14-CFD-10	-0.0776	-0.8170	0.6618	1.0000
CFD-18-CFD-10	-0.0770	-0.8164	0.6624	1.0000
CFD-2-CFD-10	-0.0184	-0.7579	0.7210	1.0000
FWD-10-CFD-10	-0.1922	-0.9316	0.5473	0.9978
FWD-14-CFD-10	-0.3047	-1.0442	0.4347	0.9306
FWD-2-CFD-10	-0.5062	-1.2456	0.2332	0.4026
FWD-20-CFD-10	-1.1304	-1.8698	-0.3910	0.0006
OWD-10-CFD-10	-0.4847	-1.2241	0.2547	0.4636
OWD-12-CFD-10	-0.4482	-1.1876	0.2913	0.5736
OWD-2-CFD-10	0.2430	-0.4964	0.9825	0.9853
CFD-18-CFD-14	0.0006	-0.7388	0.7400	1.0000
CFD-2-CFD-14	0.0592	-0.6803	0.7986	1.0000
FWD-10-CFD-14	-0.1145	-0.8540	0.6249	1.0000
FWD-14-CFD-14	-0.2271	-0.9665	0.5123	0.9913
FWD-2-CFD-14	-0.4286	-1.1680	0.3109	0.6338
FWD-20-CFD-14	-1.0528	-1.7922	-0.3134	0.0014
OWD-10-CFD-14	-0.4071	-1.1465	0.3324	0.6984
OWD-12-CFD-14	-0.3706	-1.1100	0.3689	0.7994
OWD-2-CFD-14	0.3207	-0.4188	1.0601	0.9060
CFD-2-CFD-18	0.0586	-0.6809	0.7980	1.0000
FWD-10-CFD-18	-0.1152	-0.8546	0.6243	1.0000
FWD-14-CFD-18	-0.2277	-0.9672	0.5117	0.9911
FWD-2-CFD-18	-0.4292	-1.1686	0.3102	0.6319
FWD-20-CFD-18	-1.0534	-1.7928	-0.3140	0.0014
OWD-10-CFD-18	-0.4077	-1.1471	0.3317	0.6966
OWD-12-CFD-18	-0.3712	-1.1106	0.3683	0.7978
OWD-2-CFD-18	0.3200	-0.4194	1.0595	0.9070
FWD-10-CFD-2	-0.1737	-0.9131	0.5657	0.9991
FWD-14-CFD-2	-0.2863	-1.0257	0.4531	0.9533
FWD-2-CFD-2	-0.4877	-1.2272	0.2517	0.4547
FWD-20-CFD-2	-1.1120	-1.8514	-0.3726	0.0007
OWD-10-CFD-2	-0.4662	-1.2057	0.2732	0.5185
OWD-12-CFD-2	-0.4297	-1.1692	0.3097	0.6303
OWD-2-CFD-2	0.2615	-0.4779	1.0009	0.9748
FWD-14-FWD-10	-0.1126	-0.8520	0.6269	1.0000
FWD-2-FWD-10	-0.3140	-1.0534	0.4254	0.9168
FWD-20-FWD-10	-0.9383	-1.6777	-0.1988	0.0054
OWD-10-FWD-10	-0.2925	-1.0319	0.4469	0.9463
OWD-12-FWD-10	-0.2560	-0.9954	0.4834	0.9784
OWD-2-FWD-10	0.4352	-0.3042	1.1746	0.6135
FWD-2-FWD-14	-0.2015	-0.9409	0.5380	0.9967
FWD-20-FWD-14	-0.8257	-1.5651	-0.0863	0.0196
OWD-10-FWD-14	-0.1800	-0.9194	0.5595	0.9988
OWD-12-FWD-14	-0.1434	-0.8829	0.5960	0.9998
OWD-2-FWD-14	0.5478	-0.1917	1.2872	0.2973
FWD-20-FWD-2	-0.6242	-1.3637	0.1152	0.1558
OWD-10-FWD-2	0.0215	-0.7179	0.7609	1.0000
OWD-12-FWD-2	0.0580	-0.6814	0.7974	1.0000
OWD-2-FWD-2	0.7492	0.0098	1.4887	0.0451
OWD-10-FWD-20	0.6457	-0.0937	1.3852	0.1276
OWD-12-FWD-20	0.6822	-0.0572	1.4217	0.0897
OWD-2-FWD-20	1.3735	0.6340	2.1129	0.00003
OWD-12-OWD-10	0.0365	-0.7029	0.7759	1.0000
OWD-2-OWD-10	0.7277	-0.0117	1.4672	0.0565
OWD-2-OWD-12	0.6912	-0.0482	1.4306	0.0820

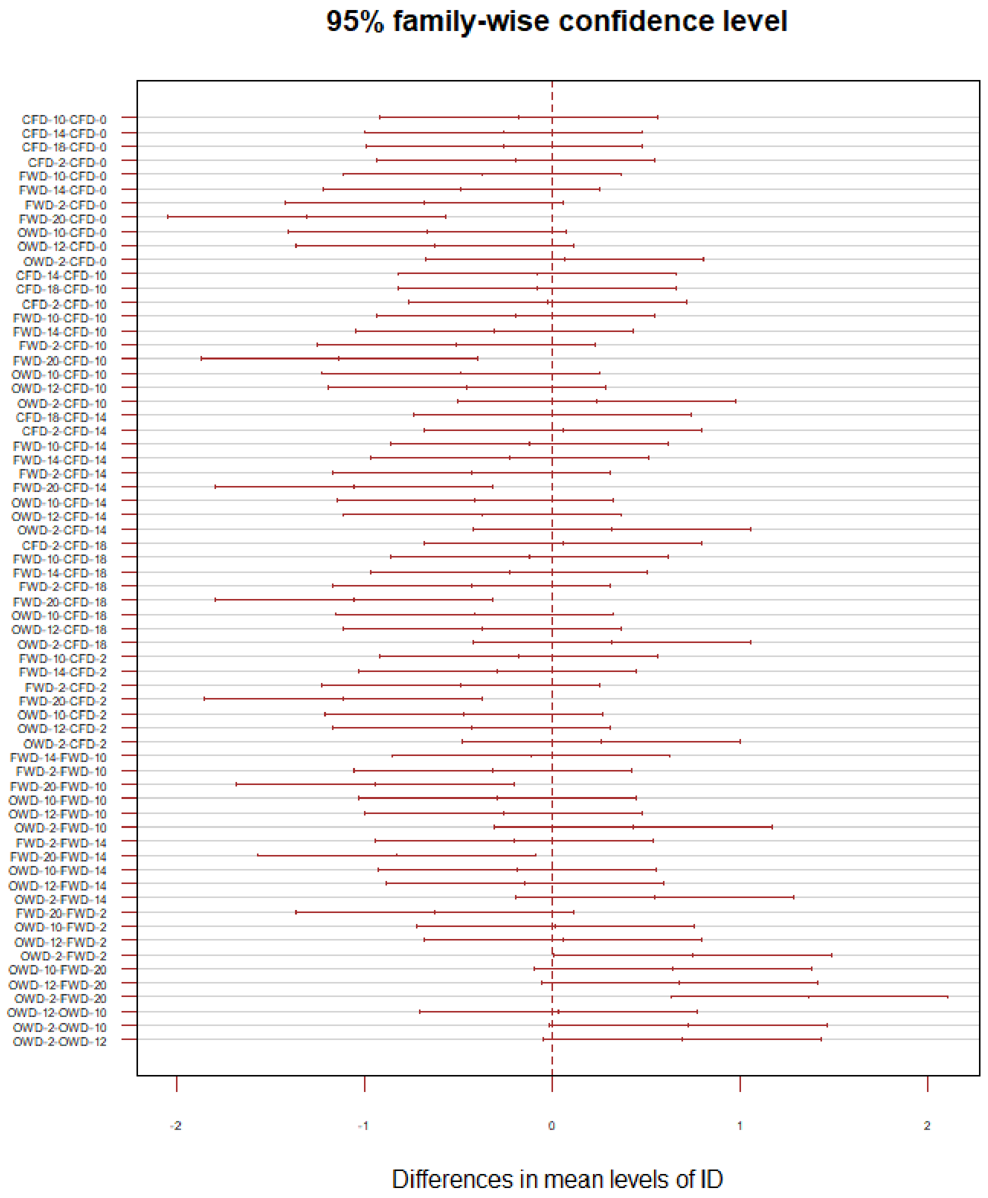

95% family-wise confidence level
CFD-10-CFD-0
CFD-14-CFD-0
CFD-18-CFD-0
CFD-2-CFD-0
FWD-10-CFD-0
FWD-14-CFD-0
FWD-2-CFD-0
FWD-20-CFD-0
OWD-10-CFD-0
OWD-12-CFD-0
OWD-2-CFD-0
CFD-14-CFD-10
CFD-18-CFD-10
CFD-2-CFD-10
FWD-10-CFD-10
FWD-14-CFD-10
FWD-2-CFD-10
FWD-20-CFD-10
OWD-10-CFD-10
OWD-12-CFD-10
OWD-2-CFD-10
CFD-18-CFD-14
CFD-2-CFD-14
FWD-10-CFD-14
FWD-14-CFD-14
FWD-2-CFD-14
FWD-20-CFD-14
OWD-10-CFD-14
OWD-12-CFD-14
OWD-2-CFD-14
CFD-2-CFD-18
FWD-10-CFD-18
FWD-14-CFD-18
FWD-2-CFD-18
FWD-20-CFD-18
OWD-10-CFD-18
OWD-12-CFD-18
OWD-2-CFD-18
FWD-10-CFD-2
FWD-14-CFD-2
FWD-2-CFD-2
FWD-20-CFD-2
OWD-10-CFD-2
OWD-12-CFD-2
OWD-2-CFD-2
FWD-14-FWD-10
FWD-2-FWD-10
FWD-20-FWD-10
OWD-10-FWD-10
OWD-12-FWD-10
OWD-2-FWD-10
FWD-2-FWD-14
FWD-20-FWD-14
OWD-10-FWD-14
OWD-12-FWD-14
OWD-2-FWD-14
FWD-20-FWD-2
OWD-10-FWD-2
OWD-12-FWD-2
OWD-2-FWD-2
OWD-10-FWD-20
OWD-12-FWD-20
OWD-2-FWD-20
OWD-12-OWD-10
OWD-2-OWD-10
OWD-2-OWD-12
-2
-1
0
1
2
Differences in mean levels of ID
